# Brain Morphology in Subacute Spinal Cord Injury: Insights Into Functional Recovery Across Brain Networks

**DOI:** 10.1002/brb3.71618

**Published:** 2026-07-23

**Authors:** Lorenzo Diana, Jothini Sritharan, Nicola Brunello, Ernst Christiaanse, Rajeev K. Verma, Giuseppe A. Zito

**Affiliations:** ^1^ Swiss Paraplegic Research Nottwil Switzerland; ^2^ Faculty of Health Sciences and Medicine University of Lucerne Lucerne Switzerland; ^3^ ARTORG Center for Biomedical Engineering Research University of Bern Bern Switzerland; ^4^ Swiss Paraplegic Centre Nottwil Switzerland; ^5^ Image Sciences Institute, Center for Image Sciences University Medical Center Utrecht Utrecht the Netherlands

**Keywords:** Spinal cord injury, functional recovery, brain morphometry, functional networks

## Abstract

**Background:**

Spinal cord injury (SCI) induces widespread structural brain reorganization extending beyond primary sensorimotor areas, yet the extent to which brain morphometry across the whole brain can inform functional recovery alongside established clinical variables remains unclear.

**Methods:**

We retrospectively analyzed 79 individuals with SCI from the SwiSCI Inception Cohort who underwent clinically acquired brain MRI during inpatient rehabilitation. The outcome measure was the Spinal Cord Independence Measure (SCIM‐III) assessed at discharge. Two cross‐validated elastic‐net regression models were evaluated: a clinical‐only model to confirm the predictive value of established clinical variables and a combined model integrating clinical variables with whole‐brain cortical gray matter volumes across different functional networks.

**Results:**

Both models achieved comparable predictive performance. However, in the combined model, a distributed set of cortical regions (including associative parietal and primary sensorimotor areas) was consistently identified by the model as potentially relevant to functional independence at discharge. Some of these regions were also significantly associated with neurological severity in the subacute stage, supporting their clinical relevance.

**Conclusion:**

In our sample, brain morphometry did not provide incremental predictive information beyond established clinical variables. Nonetheless, the combined model indicated a distributed set of cortical regions across sensorimotor and higher‐order networks showing consistent associations with functional independence at discharge, providing insights into the neural correlates of SCI recovery and candidate targets for future hypothesis‐driven neuroimaging research.

## Graphical abstract

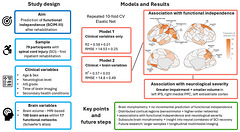

## 1 Introduction

Spinal cord injury (SCI) often results in persistent sensorimotor deficits, autonomic dysfunction, and secondary health complications, leading to marked reductions in functional independence and quality of life. (Ahuja et al. 2017; Sabariego et al. 2023) Rehabilitation outcomes after SCI are highly heterogeneous, reflecting differences in neurological severity, timing of care, and individual health profiles. (Metzger et al. 2025) Understanding the factors that contribute to functional recovery remains therefore a central challenge in neurorehabilitation research.

Prognostic models in SCI have traditionally relied on demographic and clinical variables collected early after injury. (Håkansson et al. 2024) Age, time since injury, and baseline neurological impairment assessed using the International Standards for Neurological Classification of SCI (ISNCSCI) are well‐established correlates of neurological and functional outcomes. (Kapoor and Xu 2023; Yang and Guo 2022; Belliveau et al. 2016) More recently, spine and brain imaging has emerged as promising correlate of recovery, (Ziegler et al. 2018; Seif et al. 2018; Pfyffer et al. 2024; Freund et al. 2011; Freund et al. 2013; Freund et al. 2019; Emmenegger et al. 2024; David et al. 2019) driven by the search for early biomarkers that could inform rehabilitation planning and improve participant stratification in clinical trials. (Freund et al. 2019; David et al. 2019) MRI measures of spinal cord damage, including lesion extent, (Emmenegger et al. 2024; Ding et al. 2024; Martineau et al. 2019) tissue bridges, (Pfyffer et al. 2024; Pfyffer et al. 2019; Pfyffer et al. 2021) and rostral degeneration, (Seif et al. 2018; Azzarito et al. 2020; Schading et al. 2023) have shown significant associations with long‐term neurological and functional recovery. In parallel, growing evidence indicates that SCI also induces widespread structural and functional brain changes, (Sritharan et al. 2025; Solstrand Dahlberg et al. 2018; Yu et al. 2022) already detectable in the subacute stage of injury (Ziegler et al. 2018; Freund et al. 2013; Hou et al. 2016) (i.e., from the first weeks to six months following traumatic SCI (Ahuja et al. 2017)), and progressing into the chronic phase. (Emmenegger et al. 2024; Seif et al. 2018; Diana et al. 2025)

At the brain level, the neurological consequences of SCI extend well beyond the primary sensorimotor pathways: Structural MRI studies have documented progressive gray matter reductions in prefrontal, anterior cingulate, insular, and temporal regions, areas subserving integration of multiple sensory domains, pain and emotional regulation, motivation, and cognitive functions. (Sritharan et al. 2025; Diana et al. 2025; Chen et al. 2023) These factors, together with motor and sensory functions, affect functional gains during the rehabilitation, and are associated with lower functional independence and quality of life after discharge. (Pasipanodya et al. 2021; Akar et al. 2025; Williams and Murray 2015) However, although early brain microstructural and macrostructural changes have been linked to long‐term neurological and functional outcomes after SCI, (Seif et al. 2018; Emmenegger et al. 2024; Seif et al. 2018; Huber et al. 2020) most brain‐based studies to date have focused primarily on somatosensory or motor regions, often limited by small samples sizes (although well characterized), that prevented the application of formal prediction models. (Seif et al. 2018; Freund et al. 2013; Grabher et al. 2015) It therefore remains unclear to what extent morphometric properties of brain areas spanning higher‐order brain networks, including affective and cognitive areas, can inform the recovery of functional independence following SCI.

The present retrospective study addresses this question by combining clinical data from the Swiss Spinal Cord Injury (SwiSCI) inception cohort with brain MRI data from the Swiss Paraplegic Centre (SPZ) in Nottwil, Switzerland. Our primary aim was to explore the relative contribution of brain morphometry in predicting functional independence at the end of first inpatient rehabilitation, and to identify candidate brain regions whose volumes showed consistent associations with this outcome. To this end, we estimated two cross‐validated penalized regression models: a clinical‐only model (Model 1) to confirm the predictive value of established clinical variables, and a combined model integrating clinical variables with cortical gray matter volumes of brain areas belonging to established functional networks, spanning from sensorimotor to higher‐order attentional, executive, and limbic networks (Model 2). As a secondary objective, we examined associations between brain morphometry and clinical status in the subacute stage, to better contextualize the morphometric findings within the clinical course of SCI rehabilitation.

## 2 Materials and Methods

### 2.1 Participants Selection

We retrospectively analyzed data from 79 participants with SCI enrolled in the SwiSCI inception cohort (Fekete et al. 2021) (see Section 2.2) who underwent brain MRI during their first inpatient rehabilitation at SPZ. Inclusion criteria were: age ≥18 years; availability of at least one Spinal Cord Independence Measure III (SCIM III) assessment during inpatient rehabilitation and one at discharge; and availability of a brain MRI acquired in the subacute stage of the injury (>2 weeks post‐injury (Hou et al. 2016) and up to 6 months post‐injury (Ahuja et al. 2017)), within 20 days of the clinical assessment. This threshold was defined pragmatically, together with the clinical team (E.C., R.K.V.), to accommodate the fact that in routine clinical care, neurological assessments and MRI acquisitions follow independent schedules. It was assumed that neither neurological status nor cortical grey matter morphometry, which reflects structural changes unfolding over weeks to months, would be substantially affected by short‐term clinical fluctuations within this window. Finally, participants with evidence of acquired brain injury or other neurological disorders were excluded based on clinical records and MRI inspection by a trained radiologist (R.K.V).

The use of SwiSCI data was approved by the SwiSCI Steering Committee within the framework of a SwiSCI nested project (2024‐N‐001). Written informed consent was obtained from all subjects involved in the study according to the SwiSCI cohort study. The present study was approved by the Ethics Committee of Northwest and Central Switzerland (EKNZ; ID 2025‐00222) and conducted in accordance with the Declaration of Helsinki.

### 2.2 Clinical Data From the SwiSCI Inception Cohort

Clinical data were extracted from the SwiSCI inception cohort, a multicenter cohort study conducted across four Swiss SCI rehabilitation centers (Nottwil, Basel, Sion, Balgrist).

#### 2.2.1 Primary Outcome—Functional Independence at Discharge

The primary outcome was the functional independence at discharge from rehabilitation (i.e., SCIM III total scores), ranging from 100 (complete independence) to 0 (complete dependence from external help). This was selected as the SCIM III is the gold‐standard in clinical settings, used to quantify functional independence in the activities of daily living (self‐care, respiration and sphincter management) and mobility in different indoor and outdoor contexts.

#### 2.2.2 Clinical Predictors

We identified a set of demographic and clinical variables representing each participant's subacute clinical status. For each participant, among the multiple available clinical assessments, we selected the one that was closest in time to the MRI acquisition. The following variables were selected (see the Supplementary Material for more details): age at SCI, sex, AIS grade (A‐D, reflecting the severity of sensorimotor impairment), neurological level of injury (NLI), SCIM III total score at the subacute stage (SCIM_sub), time since injury (TSI), and cumulative comorbidity burden assessed with the Cumulative Illness Rating Scale (CIRS).

### 2.3 Neuroimaging Processing

Brain MRI data acquired between 2014 and 2024 were obtained on a Philips Achieva 3T scanner. Due to heterogeneous clinical acquisition protocols, all scans were harmonized using SynthSR to generate 1‐mm isotropic T1‐weighted images (Iglesias et al. 2023; Iglesias et al. 2021) This approach has shown highly robust results, and has been successfully applied in recent clinical studies. (Baldi et al. 2025; Achiron et al. 2025) Images were processed using CAT12 (v12.9) (Gaser et al. 2024) implemented in SPM12 running in MATLAB R2019b. Following a previous work in chronic SCI, (Diana et al. 2025) GM volumes were extracted using the 100‐region Schaefer cortical parcellation, (Yeo et al. 2011) organized into major functional networks, i.e., the somatomotor network (SMN), the central and peripheral visual networks (VCN and VPN), the ventral and dorsal attentional networks (VAN and DAN), the executive control network (ECN), the limbic network, and the Default Mode Network (DMN). More details about neuroimaging processing are reported in the Supplementary Materials.

### 2.4 Analyses

Analyses were performed in RStudio 2025.05.1, (RStudio 2020) with specific R packages (see the Supplementary Materials). Changes in SCIM scores between the subacute stage and discharge were assessed using a paired *t*‐test, under the assumption of normality (Shapiro‐wilk test: *W* = 0.99, *p* = 0.97). Missing CIRS score (6/79) were imputed with Multiple Imputation by Chained Equations (MICE) (van Buuren and Groothuis‐Oudshoorn 2011) with predictive mean matching (PMM) and 20 imputed datasets, under the missing‐at‐random assumption. (Fekete et al. 2022) All clinical variables (age at injury, sex, AIS, NLI, TSI, and SCIM_sub) were used as predictors in the imputation model to ensure that the imputed CIRS values reflected meaningful clinical patterns.

#### 2.4.1 Prediction of Functional Independence

The primary objective of this study was to explore whether subacute brain morphometry across different functional networks provides information about functional recovery complementary to established clinical variables. To address this question, we constructed two models to predict SCIM score at discharge: Model 1, a clinical model including only clinical predictors (age, sex, AIS, NLI, SCIM_sub, TSI, and CIRS), and Model 2, integrating the full set of clinical variables with 100 cortical gray matter (GM) volume predictors from the Schaefer atlas.

Model development followed a penalized regression framework. Specifically, due to the large number of correlated brain predictors, we used penalized regression with an elastic net penalty, which combines L1 (lasso) and L2 (ridge) regularization. (Wu et al. 2022; Teipel et al. 2017) Elastic net was preferred over pure LASSO due to the substantial multicollinearity among cortical measures. (Zou and Hastie 2005) All predictors were standardized. The alpha parameter was fixed at 0.5, balancing sparsity and coefficient shrinkage, and avoiding additional tuning complexity. The optimal lambda was determined within the 10‐fold cross‐validation procedure (see below) by selecting the value that minimized the mean cross‐validated error, as implemented in the *glmnet* package.

Model performance was assessed through repeated 10‐fold cross‐validation. For each repetition, the dataset was randomly partitioned into 10 folds; in each fold, 9 folds were used for training and the remaining fold for testing. This procedure was repeated 100 times for each model to obtain stable estimates of the coefficient of determination (R^2^) and the root mean squared error (RMSE). Mean and standard deviation (SD) of R^2^ and RMSE across repetitions were also computed. For each predictor, mean coefficient values, their standard deviations, and frequency of selection were calculated to summarize their contribution to the model.

To assess the potential influence of MRI sequence type on our findings, a supplementary analysis was conducted by adding a binary predictor (native T1 vs. FLAIR) to both models. Results are reported in the Supplementary Materials.

#### 2.4.2 Association Between Clinical Variables and Brain Morphometry

As a secondary, exploratory objective, we examined the associations between brain morphometry and participants’ clinical status in the subacute stage, using the same clinical variables included in the previous models. Specifically, for the most consistently selected brain regions of Model 2 (here defined as those > 50% of selection frequency), we ran a general linear model (GLM) with the regional GM volume as the outcome variable and the following clinical predictors: NLI, AIS, TSI, and CIRS. Age, sex, SCIM_sub, and total intracranial volume (TIV) were included as covariates of no interest to account for known demographic and anatomical influences on brain morphology. Given the limited number of examined regions and the exploratory nature of these analyses, no correction for multiple comparisons was applied.

## 3 Results

As reported in Table 1, the sample included 79 participants with an average age at the time of SCI of 45.7 ± 15.8 years. The majority sustained traumatic injuries (N = 74), resulting in complete (AIS A; N = 35) or incomplete (AIS B–D; N = 44) neurological damage across a range of NLI. The average rehabilitation length was 198.3 ± 60.3 days, and varied substantially according to individual clinical needs.

**TABLE 1:** Demographic and clinical characteristics of the sample.

Variable	Value
Age (years)	45.7 ± 15.8 years (20–81)
Sex (F/M)	(11 F / 68 M)
NLI	32 cervical / 35 thoracic / 12 lumbar
AIS	35 A / 9 B / 11 C / 24 D
Etiology	74 traumatic / 5 non‐traumatic (3 vascular, 2 infections)
Rehabilitation Length (days)	198.3 ± 60.3 (62–342)
SCIM_sub	34.7 ± 20.5 (1–81)
TSI (days)	50.22 ± 31.9 (11–150)
TSI—MRI (days)	54.37 ± 26.03 (17–130)

*Note*: Continuous variables are reported as mean ± SD and range is reported in brackets. AIS_sub = American Spinal Injury Association Impairment Scale at baseline (i.e., at the subacute assessment during inpatient rehabilitation); NLI_sub = Neurological level of injury at baseline (i.e., at the subacute assessment during inpatient rehabilitation); TSI = time since injury (i.e., days from SCI to subacute assessment); TSI—MRI = time since injury to brain Magnetic Resonance Imaging in days; SCIM_sub = Spinal Cord Independence Measure III at baseline (i.e., at the subacute assessment during inpatient rehabilitation).

**FIGURE 1:**
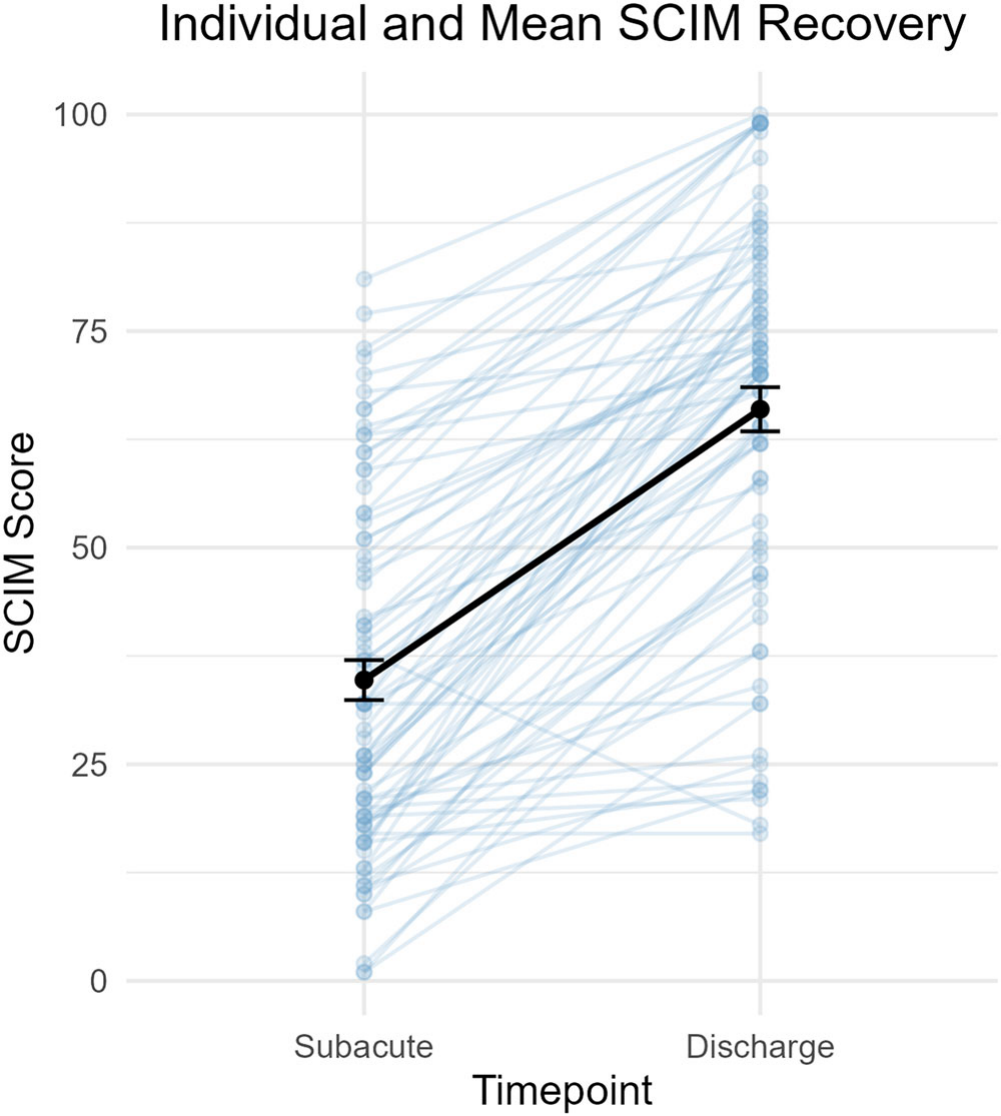
Individual and mean scores of Spinal Cord Independence Measure (SCIM) III in the subacute stage and at discharge. Error bars depict the standard error.

As expected, we observed a significant increase in the SCIM III score at discharge (Mean ± SD = 65.97 ±22.65; *t_78_* = 15.39, *p* < 0.001), compared to the SCIM III score in the subacute stage (34.72 ± 20.51, Figure 1).

### 3.1 Prediction of Functional Independence

Across the repetitions of the 10‐fold CV, the model including only clinical variables (Model 1) achieved a mean (± SD) R^2^ = 0.58 ± 0.01 and a mean RMSE = 14.53 ± 0.25, showing moderate out‐of‐sample predictive performance (see Table 2). The strongest predictor was AIS, with a mean coefficient (coeff) = 6.08, indicating that participants with more severe neurological impairment (greater lesion completeness) experienced lower functional recovery. Other predictors contributed to a lesser extent, suggesting that participants who achieved higher functional independence at discharge tended to be younger (Age, mean coeff = −0.222), had a lower NLI (mean coeff = 0.687), demonstrated greater functional independence at the initial subacute assessment (SCIM_sub, mean coeff = 0.505), and were less affected by secondary health conditions (mean coeff = −0.180).

**TABLE 2:** Clinical predictors of functional independence at discharge—Model 1.

Predictor	Mean Coeff	SD Coeff	Frequency of selection
AIS	6.08	0.55	100
NLI	0.69	0.08	100
Age	−0.22	0.05	100
TSI	−0.20	0.02	100
SCIM_sub	0.51	0.03	100
CIRS	−0.18	0.11	89.9
Sex	−1.60	1.78	67.1

*Note*: predictors are sorted by frequency of selection and magnitude of the mean coefficient. AIS_sub = American Spinal Injury Association Impairment Scale at the subacute assessment; CIRS = Cumulative Illness Rating Scale; NLI_sub = Neurological level of injury at the subacute assessment; SCIM_sub = Spinal Cord Independence Measure III at the subacute assessment; TSI = time since injury.

**FIGURE 2:**
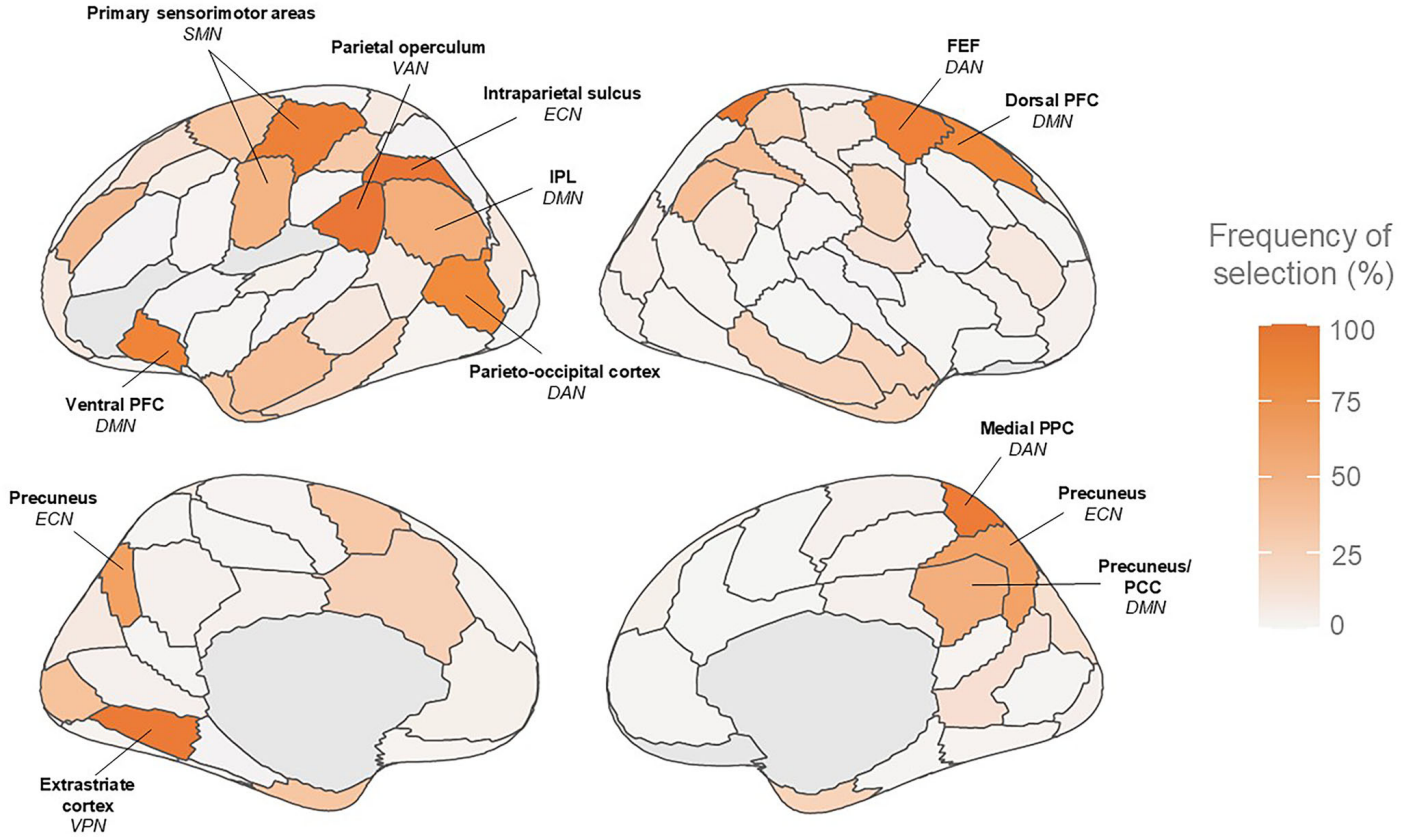
Frequency of selection of brain regions in Model 2. Brain regions are colored based on their frequency of selection for Model 2 (i.e., darker color = highest frequency of selection across the repeated estimations of the model). Regions with frequency of selection > 50% across 1000 cross‐validation folds are labeled. DMN = default mode network; DAN = dorsal attentional network; ECN = executive control network; FEF = frontal eye field; IPL = inferior parietal lobule; PCC = posterior cingulate cortex; PFC = prefrontal cortex; PPC = posterior parietal cortex; SMN = sensorimotor network; VAN = ventral attentional network; VPN = visual peripheral network.

Model 2, which integrated clinical and morphometric variables, achieved similar predictive performance (R^2^ = 0.57 ± 0.03; RMSE = 14.8 ± 0.49) but pointed to potential brain structural correlates of functional independence at discharge. Specifically, alongside AIS (mean coefficient = 4.12; see supplementary Table S1), which remained the strongest clinical predictor, several areas of the sensorimotor and non‐sensorimotor areas across bilateral functional networks were consistently identified across cross‐validation folds (Figure 2). Among the areas showing the highest selection frequency (see Table 3 and Supplementary Table S1 for the complete list), associations between GM volume and functional recovery were observed in: the left parietal operculum within the VAN (mean coefficient = 7.47), the left intraparietal sulcus (mean coefficient = 6.88), the right medial posterior parietal cortex within the DAN (mean coefficient = 3.51), the left primary sensorimotor area (mean coefficient = 2.73), as well as dorsal (mean coefficient = −2.26) and ventral areas of the prefrontal cortex (mean coefficient = −2.42) within the DMN.

**TABLE 3:** Most frequently selected brain areas for Model 2.

Predictor	Mean Coeff	SD Coeff	Frequency of selection
Left parietal operculum—VAN	7.42	1.71	100
Left IPS—ECN	6.89	1.96	99.8
Left extrastriate cortex—VPN	−5.03	2.08	98.3
Right medial PPC—DAN	3.47	1.67	97.2
Left primary SM area (dorsal)—SMN	2.7	1.62	91.6
Right FEF—DAN	−1.87	1	89.8
Left ventral PFC—DMN	−2.38	1.42	89
Right dorsal PFC—DMN	−2.31	1.52	83.4
Left parieto‐occipital cortex—DAN	−2.3	1.75	80.4
Left precuneus—CEN	−3.72	3	65.2
Right precuneus—CEN	−2.17	1.61	62.5
Left IPL—DMN	0.85	0.69	53.1
Right precuneus/PCC—DMN	−1.77	1.69	52.2

*Notes*: brain areas are sorted by frequency of selection and magnitude of the mean coefficient. For visualization purposes, only brain areas with a frequency of selection > 50% are reported. The complete list can be found in the supplementary materials. Areas according to the Schaefer atlas labeling: Left parietal operculum—VAN = lSalVentAttnA_ParOper_1; Left IPS—ECN = lContA_IPS_1; Left extrastriate cortex—VPN = lVisPeri_ExStrInf_1; Left precuneus—CEN = lContC_pCun_1; Right medial PPC—DAN = rDorsAttnB_PostC_2; Left primary SM area (dorsal)—SMN = lSomMotA_1; Left ventral PFC—DMN = lDefaultB_PFCv_1; Left parieto‐occipital cortex—DAN = lDorsAttnA_ParOcc; Right dorsal PFC—DMN = rDefaultA_PFCd_1; Right precuneus—CEN = rContC_pCun_1; Right FEF—DAN = rDorsAttnB_FEF_1; Right precuneus/PCC—DMN = rDefaultA_pCunPCC_1; Left primary SM area (ventral)—SMN = lSomMotB_Cent_1;.

Left IPL = lDefaultB_IPL_1. CEN = control executive network; DMN = default mode network; DAN = dorsal attentional network; ECN = executive control network; FEF = frontal eye field; IPL = inferior parietal lobule; IPS = intraparietal sulcus; PCC = posterior cingulate cortex; PFC = prefrontal cortex; PPC = posterior parietal cortex; SM = sensorimotor; SMN = sensorimotor network; VAN = ventral attentional network; VPN = visual peripheral network.

### 3.2 Association Between Brain Morphology and Clinical Predictors

The GLMs revealed a positive association between the severity of sensorimotor impairment in the subacute stage and GM volume. Specifically, greater lesion completeness (AIS) was linked to smaller GM volume in the left intraparietal sulcus (IPS; *β* = 0.13, *p* = 0.005). Similarly, higher NLI was associated with reduced volume in the medial PPC (*β* = 0.02, *p* = 0.028) and the left extrastriate cortex (*β* = 0.02, *p* = 0.04).

## 4 Discussion

This study investigated whether brain morphometric properties across different functional networks, acquired during the subacute stage of SCI, provide parallel information to standard clinical variables in explaining functional independence at discharge from first inpatient rehabilitation.

Using a cross‐validated elastic‐net framework, we evaluated two models: one based solely on clinical predictors, and one combining clinical and brain measures. Three main findings emerged. First, clinical variables alone predicted functional independence at discharge with moderate accuracy—consistent with extensive prior literature (e.g., see (Håkansson et al. 2024) for a review). Second, the addition of brain morphometry did not provide incremental information in terms of predictive accuracy, but pointed to a distributed set of cortical regions across different brain networks whose structural characteristics showed consistent associations with the outcome. Third, exploratory analyses showed that some of these brain areas were also associated with the clinical status of participants in the subacute stage, suggesting that brain morphology in these regions may capture meaningful variation linked to neurological severity.

### 4.1 Structural Brain Changes Across Functional Networks

To our knowledge, this is the first study to evaluate whether brain morphology, especially from a functionally informed, network‐based perspective, can inform the prediction of functional recovery in SCI. Previous SCI prediction studies have focused predominantly on spinal cord imaging, (Håkansson et al. 2024) either via global indices such as the Brain and Spinal Injury Center (BASIC) score (Sizheng et al. 2022) or quantitative lesional metrics such as maximum cord compression, (Yan et al. 2023) or the width of tissue bridges. (Pfyffer et al. 2024) Where brain imaging has been considered, studies have largely focused on primary somatosensory and motor pathways, (Seif et al. 2018; Emmenegger et al. 2024; Seif et al. 2018; Chen et al. 2023) leaving open the question of whether structural changes in higher‐order cortical networks carry independent prognostic information.

Although in our sample the addition of brain morphometry variables did not improve the ability of clinical variables to predict functional recovery on unseen data, several cortical areas spanning sensorimotor and higher‐order networks showed consistent associations with functional independence at discharge. The volume of different sensorimotor cortices (in particular the left primary sensorimotor areas) was among the most frequently selected predictors, i.e., smaller gray‐matter volume in these regions was associated with poorer functional gains. This pattern is consistent with previous studies reporting regional atrophy in primary sensorimotor motor areas following SCI and its association with long‐term neurological and functional outcomes. (Ziegler et al. 2018; Freund et al. 2013; Yu et al. 2022; Huber et al. 2020) This also aligns with known mechanisms of SCI‐related neurodegeneration, including myelin loss and iron accumulation, (David et al. 2019) that contribute to structural decline in primary somatosensory cortices and subcortical structures. Nonetheless, multiple regions within the posterior parietal cortex (PPC) also emerged as frequently selected predictors. For instance, lower GM volume in the left parietal operculum, the left IPS, and the right medial PPC, was associated with poorer functional independence at discharge. These regions form a multimodal integration hub supporting both sensory and higher cognitive functions, and are tightly connected to premotor and motor pathways. (Whitlock 2017) The left IPS, known for its role on higher attentional processes and executive controls, may also contribute to motor recovery, as recently shown in chronic stroke. (Salazar et al. 2024; D'Imperio et al. 2021) Specifically, through its connectivity within the DAN and sensorimotor networks, the IPS may support the integration of cognitive and motor functions during rehabilitation, thus promoting functional recovery. Likewise, the right medial PPC, plays a well‐established role in mediating attention and motor planning. (Breveglieri et al. 2025) Importantly, both areas showed significant associations with the severity of sensorimotor impairment (AIS grade and NLI, respectively), further supporting the role of the PPC in the prediction of functional recovery. Interestingly, the parietal operculum was also consistently identified across cross‐validation folds. This area is strongly connected with primary somatosensory cortices, thalamic nuclei conveying sensorimotor information, and parietal networks involved in multisensory integration, (Eickhoff et al. 2010) thus contributing to the integration of sensorimotor and proprioceptive information, processes that can be disrupted following SCI and may affect rehabilitation and functional recovery.

An aspect of our findings that warrants further investigation concerns the regions showing negative associations between gray matter volume and functional recovery. While most positive associations (smaller volume → poorer recovery) are consistent with possible degenerative changes, we also observed negative associations (bigger volume → poorer recovery). One speculative explanation is that larger volumes in these regions reflect early compensatory reorganization in individuals with more severe neurological deficits, a structural response that may not translate into functional gains within the rehabilitation period. It is worth noting that morphological brain changes following SCI are not only associated with reductions of brain volume: For instance, prior work in chronic SCI has documented increases in cortical volume in the precuneus after the injury, (Diana et al. 2025) potentially reflecting adaptive response in implementing and consolidating new coordinated motor schemes. Similarly, in our study, right precuneus volume was negatively associated with functional recovery. Likewise, frontal regions across dorsal and ventral networks showed a potential contribution to prediction; their known roles in executive control, attention, and action planning suggest they may support adaptive cognitive strategies during rehabilitation. (Song 2019)

Taken together, these findings align with previous evidence about widespread structural (Yu et al. 2022) and functional reorganization (Sritharan et al. 2025) across multiple cortical networks. Such a distributed pattern suggests that functional recovery may depend on the integrity of networks supporting sensorimotor adaptation, attentional orienting, and cognitive control during rehabilitation. However, the mechanisms underlying structural brain changes following SCI, particularly in higher‐order areas, remain incompletely understood. While changes in primary sensorimotor areas have been mostly explained as progressive atrophy due to anterograde and trans‐synaptic degeneration, (Freund et al. 2019; David et al. 2019) structural changes in higher‐order brain areas could likely reflect a combination of indirect mechanisms acting in parallel.

First, deafferentation of sensorimotor cortices may affect both structurally and functionally connected areas, responsible for higher order functions, such as the fronto‐parietal areas emerged in this study. Indeed, this explanation is consistent with changes in resting‐state functional connectivity between areas of the somatomotor network and other networks, such as the VAN, documented after SCI. (Sritharan et al. 2025) Second, SCI triggers a sustained neuroinflammatory response that extends well beyond the injury site: experimental evidence demonstrates chronic microglial activation in the hippocampus, thalamus, and cerebral cortex following isolated spinal cord contusion, driven in part by the distal release of neuroactive signaling molecules that propagate the inflammatory cascade to remote brain structures. (Wu et al. 2014) Third, neuropathic pain, which affects the majority of people with SCI, (Burke et al. 2017) could be itself an independent driver of structural brain reorganization, with gray matter reductions consistently reported in pain‐modulatory regions including the anterior cingulate cortex, insula, thalamus, and dorsolateral prefrontal cortex in individuals with SCI. (Grabher et al. 2015) Finally, as highlighted by Dahlberg and colleagues (Solstrand Dahlberg et al. 2018), indirect factors including motor disuse, altered behavior, psychological distress, and the neurological effects of long‐term medication use may further compound structural remodeling in regions not directly affected by the primary injury.

### 4.2 Limitations

Our study has some limitations that should be acknowledged.

First, the absence of incremental predictive gain in Model 2 warrants discussion. With 79 participants and over 100 brain morphometric predictors, the available sample may be insufficient to reliably detect the marginal contribution of brain variables over and above strong clinical predictors. Moreover, variable selection reliability may be limited under these conditions; To mitigate this, we adopted a repeated CV approach reporting mean coefficient estimates, standard deviations, and selection frequencies across different fold‐fits, providing a transparent characterization of selection variability—though we acknowledge this does not fully resolve the instability inherent to high‐dimensional selection with small samples.

Additionally, the variability in timing of clinical and MRI assessments could introduce noise into both clinical and morphometric measures, potentially attenuating associations that might otherwise emerge. In this regard, we aimed to pair clinical and radiological information as closely as possible, and we retained this variability, by including TSI as a covariate in all models to mitigate its potential confounding influence. Nonetheless, this variability mirrors the real‐world conditions of inpatient rehabilitation, arguably making our findings representative of routine clinical practice. Adequately powered prospective studies with standardized imaging protocols are needed to determine whether the candidate regions identified here carry actual incremental prognostic value.

A further sample‐related limitation is the sex imbalance of our sample. Although male predominance in SCI is well‐documented epidemiologically, (Fekete et al. 2021) evidence on the impact of sex on neurological and functional recovery is mixed. (Furlan et al. 2025; Sipski et al. 2004; Scivoletto et al. 2004) Notably, women with SCI may be more susceptible to pain, (Norrbrink Budh et al. 2003) which is itself a driver of structural brain reorganization, (Jutzeler et al. 2016) and sex‐based differences in gray matter volume in the general population have been linked to modulatory effects of sex hormones. (Witte et al. 2010) Future studies with larger and more balanced samples should systematically examine sex as a biological variable in brain morphometry and rehabilitation outcomes after SCI.

Moreover, although some brain morphometric measures showed associations with functional independence and neurological severity, they may not capture the full range of neurobiological processes relevant to post‐SCI recovery. Microstructural integrity, functional connectivity, and spinal cord imaging metrics reflect complementary aspects of neuroplasticity and degeneration after SCI, and should be integrated in future studies. (Freund et al. 2019) In our retrospective design, however, a comprehensive and temporally aligned set of multimodal brain–spinal cord acquisitions was not available, and imaging data had to be harmonized across multiple clinical MRI protocols using SynthSR; while this approach was specifically designed to enable morphometric analyses across heterogeneous sequences, (Iglesias et al. 2023; Iglesias et al. 2021) the synthesis process may introduce residual variability that native T1‐weighted acquisitions would not. Finally, the availability of a single brain MRI per participant represents an additional limitation. The lack of longitudinal neuroimaging limits a direct interpretation of regional volume differences as dynamic brain changes over time and their relationship with functional recovery.

### 4.3 Future Prospectives

Although the present findings do not yet support clinical translation, they may carry concrete implications for future research. The cortical regions identified provide an empirically derived set of regions of interest for future prospective studies, enabling a shift from the data‐driven approach employed here toward confirmatory designs in adequately powered cohorts. Such studies would ideally integrate structural morphometry with functional connectivity and diffusion imaging to establish whether the observed structural differences translate into disrupted network function mediating rehabilitation outcomes. Finally, the identification of fronto‐parietal regions as structural correlates of recovery raises the hypothesis that non‐invasive brain stimulation, for which preliminary efficacy has been shown in SCI for motor recovery, neuropathic pain, and mood, (Chen et al. 2023; Li et al. 2021) could serve as a rehabilitation adjunct targeting these networks.

## 5 Conclusion

In the present study, brain morphometry did not improve the prediction of functional independence at discharge beyond standard clinical variables. Nonetheless, the combined model pointed to a distributed set of cortical regions spanning sensorimotor and higher‐order networks that showed consistent associations with functional independence at discharge, some of which also correlated with neurological severity in the subacute stage. These findings underscore the complexity of recovery mechanisms following SCI and support further investigation of brain morphometry as a neuroimaging tool for understanding functional outcomes. Future research in larger samples, integrating multimodal spinal and brain imaging, will be essential to determine how neuroimaging can most effectively support individualized prediction and rehabilitation planning in SCI.

## Author Contributions

**Nicola Brunello**: Writing – review and editing. **Jothini Sritharan**: writing – review and editing. **Rajeev K. Verma**: conceptualization, funding acquisition, project administration, supervision, writing – review and editing. **Ernst Christiaanse**: writing – review and editing. **Giuseppe A. Zito**: conceptualization, methodology, funding acquisition, project administration, writing – review and editing, supervision. **Lorenzo Diana**: conceptualization, investigation, formal analysis, methodology, visualization, writing – original draft, writing – review and editing.

## Funding

The authors have nothing to report.

## Ethics Statement

The use of SwiSCI data was approved by the SwiSCI Steering Committee within the framework of a SwiSCI nested project (2024‐N‐001). Written informed consent was obtained from all subjects involved in the study according to the SwiSCI cohort study. The present study was approved by the Ethics Committee of Northwest and Central Switzerland (EKNZ; ID 2025‐00222) and conducted in accordance with the Declaration of Helsinki.

## Supporting information




**Supplementary Material**: brb371618‐sup‐0001‐SuppMat.docx

## Data Availability

Data will be made available upon request to the corresponding author.
