# Supplementary material for: Brain Morphology in Subacute Spinal Cord Injury: Insights Into Functional Recovery Across Brain Networks
Source: Brain Behav. 2026 Jul 23;16(7):e71618. doi: 10.1002/brb3.71618 (PMC13396882; doi:10.1002/brb3.71618)
Supplement: Supplementary file 1 — Supplementary Material: brb371618‐sup‐0001‐SuppMat.docx [file BRB3-16-e71618-s001.docx]

**Supplementary Material**

***Clinical predictors***

We identified a set of demographic and clinical variables representing each participant’s subacute clinical status. For each participant, we selected the timepoint that was closest in time to the MRI acquisition.

The following variables were used as clinical predictors:

• age at the time of SCI (years) and sex;

• the ASIA impairment in the subacute stage (AIS), rating the type of injury into categories depending on the completeness of the sensorimotor deficit, namely, A (complete motor and sensory loss), B (sensory incomplete loss), C , and D (motor incomplete loss). For the following analyses, AIS was treated as an ordinal variable with A = 1, B = 2, C = 3, and D = 4, reflecting decreasing neurological severity;

• the neurological level of injury in the subacute stage (NLI), defined as the most caudal segment of the spinal cord with intact sensorimotor functions; the NLI values could range from 1 (C1 level) to 30 (intact sensorimotor functions). In case of discrepancy between the most caudal intact section between Left and Right Sensory and Motor levels, the most rostral NLI was chosen ^1^;

• the SCIM III total score in the subacute stage (SCIM_sub); the total score ranges from 0 (complete dependence) to 100 (complete independence).

• the time since injury (TSI), i.e., the number of days from the SCI and the functional assessment with the SCIM III in the subacute phase, included to account for differences in the timing of subacute evaluations;

• the Cumulative Illness Rating Scale (CIRS) total score collected at T4 was also included to quantify the burden of secondary health conditions emerging during the rehabilitation, which could affect functional recovery. The CIRS assesses the severity of medical problems across 14 different domains/organ systems, namely, cardiac, hypertension, vascular, respiratory, EENT (Eye, Ear, Nose, Throat), gastrointestinal, hepatic, renal, genitourinary, musculoskeletal, neurological, endocrine-metabolic, and psychiatric/behavioral. The cumulative score ranges from 0 (absence of clinical conditions) to 56 (extremely severe impairment across multiple domains).

***Neuroimaging Processing***

We considered neuroimaging data acquired at the Radiology Department of the SPZ between 2014 to 2024. Because the available brain scans were heterogeneous (largely acquired as part of routine clinical examinations) and high-resolution T1-weighted structural images suitable for morphometric analysis were not always available, we adopted a harmonization strategy. We first selected the most consistently available sequence type across participants, namely, non-isotropic T2-weighted FLAIR (35/79), 3D isotropic T2-weighted FLAIR (36/79), high resolution (1mm) isotropic T1-weighted (5/79), or low-resolution (2mm) isotropic T1-weighted (3/79). All scans were acquired using a Philips Achieva 3T MRI scanner (release: 5.4.1; Philips Healthcare, Best, Netherlands). All scans were converted into 1mm isotropic MPRAGE-like images using SynthSR, a convolutional neural network designed to synthesize T1-weighted structural images from diverse MRI contrasts while standardizing geometry and resolution.^2, 3^ This approach has shown highly robust results, and has been successfully applied in recent clinical studies.^4, 5^ The synthesized T1-weighted images were processed using the Computational Anatomy Toolbox (CAT12, v12.9)^6^ implemented in SPM12 (Wellcome Centre for Human Neuroimaging, University College London, UK) running under MATLAB R2019b (The MathWorks Inc., USA). The default CAT12 morphometry pipeline was used to estimate regional GM volumes. Preprocessing with CAT12 was performed as follows: Anatomical scans were denoised, bias-corrected, and spatially resampled, followed by tissue segmentation into GM, white matter, and cerebrospinal fluid using CAT12 tissue probability maps. Partial volume estimation was applied, and images were normalized to the Montreal Neurological Institute (MNI) template using CAT12’s geodesic shooting algorithm.^7^ Following our previous work in chronic SCI,^8^ regional GM volumes were extracted from the Schaefer et al.^9^ local–global cortical parcellation (100-region version), an atlas derived from multimodal functional connectivity and structural features in 1,489 subjects. This parcellation provides 100 cortical regions (50 per hemisphere) organized into major functional networks, i.e., the somatomotor network (SMN), the central and peripheral visual networks (VCN and VPN), the ventral and dorsal attentional networks (VAN and DAN), the executive control network (ECN), the limbic network, and the Default Mode Network (DMN).

***R packages***

The following R packages were used:

- Wickham H, Bryan J. readxl: Read Excel Files. 2025.
- Wickham H, François R, Henry L, Müller K, Vaughan D. dplyr: A Grammar of Data Manipulation. 2023.
- Wickham H. ggplot2: Elegant Graphics for Data Analysis: Springer-Verlag New York; 2016.
- Wickham H, Averick M, Bryan J, Chang W, McGowan LD, François R, et al. Welcome to the tidyverse. Journal of Open Source Software. 2019;4(43):1686.
- Friedman J, Hastie T, Tibshirani R. Regularization Paths for Generalized Linear Models via Coordinate Descent. Journal of Statistical Software. 2010;33(1):1-22.
- Venables WN, Ripley BD. Modern Applied Statistics with S. Fourth ed. New York: Springer; 2002.
- van Buuren S, Groothuis-Oudshoorn K. mice: Multivariate Imputation by Chained Equations in R. Journal of Statistical Software. 2011;45(3):1-67.
- Juergen G, Uwe L. nortest: Tests for Normality. 2015.
- Athanasia M. Mowinckel and Didac Vidal-Piñeiro (2019). Visualisation of Brain Statistics with R-packages ggseg and ggseg3d. arXiv:1912.08200

**Results**

**Table S1. Model 2 – Frequency of selection of clinical and brain predictors**

| **Predictor** | **Mean Coeff** | **SD**  **Coeff** | **Frequency of selection** |
| --- | --- | --- | --- |
| **AIS** | 4.12 | 0.507 | 100 |
| **CIRS** | -0.491 | 0.163 | 100 |
| **NLI** | 0.627 | 0.131 | 100 |
| **TSI** | -0.152 | 0.0207 | 100 |
| lSalVentAttnA_ParOper_1 | 7.42 | 1.71 | 100 |
| **SCIM_sub** | 0.461 | 0.0316 | 100 |
| lContA_IPS_1 | 6.89 | 1.96 | 99.8 |
| lVisPeri_ExStrInf_1 | -5.03 | 2.08 | 98.3 |
| rDorsAttnB_PostC_2 | 3.47 | 1.67 | 97.2 |
| lSomMotA_1 | 2.7 | 1.62 | 91.6 |
| rDorsAttnB_FEF_1 | -1.87 | 1 | 89.8 |
| lDefaultB_PFCv_1 | -2.38 | 1.42 | 89 |
| lDorsAttnA_ParOcc_1 | -2.31 | 1.52 | 83.4 |
| rDefaultA_PFCd_1 | -2.3 | 1.75 | 80.4 |
| lContC_pCun_1 | -3.72 | 3 | 65.2 |
| rContC_pCun_1 | -2.17 | 1.61 | 62.5 |
| lDefaultB_IPL_1 | 0.853 | 0.689 | 53.1 |
| rDefaultA_pCunPCC_1 | -1.77 | 1.69 | 52.2 |
| lSomMotB_Cent_1 | -1.83 | 1.49 | 47.6 |
| lSalVentAttnB_PFCl_1 | -0.8 | 0.721 | 45.4 |
| rContA_IPS_1 | 0.986 | 0.873 | 40.9 |
| rContB_IPL_1 | 0.983 | 0.897 | 40.3 |
| lDefaultB_Temp_1 | 0.705 | 0.645 | 38.4 |
| lVisCent_Striate_1 | 1.33 | 0.889 | 36 |
| lDorsAttnB_PostC_2 | 2.9 | 2.59 | 33.7 |
| lDorsAttnB_FEF_1 | 0.924 | 0.787 | 33.1 |
| lLimbicA_TempPole_1 | 0.626 | 0.452 | 32.7 |
| lSalVentAttnA_FrMed_1 | -1.68 | 1.47 | 31.8 |
| rSomMotA_3 | 2.01 | 2.02 | 29.2 |
| lSalVentAttnB_PFCmp_1 | 1.43 | 1.25 | 27.7 |
| rTempPar_1 | 0.846 | 0.754 | 27.6 |
| lLimbicA_TempPole_2 | 0.503 | 0.428 | 26.1 |
| rLimbicA_TempPole_1 | 0.429 | 0.378 | 25.3 |
| rContB_Temp_1 | 0.654 | 0.565 | 23.4 |
| rSomMotB_Cent_1 | 1.69 | 1.59 | 23.3 |
| lDefaultA_PFCd_1 | 1.29 | 1.17 | 16.5 |
| **Sex** | -1.3 | 1.7 | 16 |
| rSomMotB_S2_2 | 1.54 | 1.54 | 14.5 |
| rVisPeri_ExStrSup_1 | 0.739 | 0.8 | 13.8 |
| rVisPeri_ExStrInf_1 | 0.956 | 0.833 | 12 |
| **Age** | -0.0281 | 0.0276 | 10.2 |
| lDefaultB_Temp_2 | -1.52 | 1.23 | 9.4 |
| lContB_PFClv_1 | 0.901 | 0.807 | 8.6 |
| rContA_PFCl_1 | -0.5 | 0.529 | 8.4 |
| rSomMotA_2 | 2.84 | 2.71 | 8.1 |
| lVisCent_ExStr_3 | 1.22 | 1.41 | 7.8 |
| lVisPeri_ExStrSup_1 | -2.61 | 2.25 | 7.7 |
| lDorsAttnB_PostC_3 | 1.91 | 1.66 | 7.4 |
| rDefaultA_IPL_1 | 1.41 | 1.13 | 7.4 |
| rSalVentAttnB_IPL_1 | -1.5 | 1.67 | 6.5 |
| rVisCent_ExStr_3 | 0.481 | 0.748 | 6 |
| lTempPar_1 | -1.09 | 1.45 | 5.2 |
| rContB_PFClv_1 | -0.475 | 0.513 | 5.1 |
| rDorsAttnB_PostC_1 | -1.24 | 1.19 | 5.1 |
| lDefaultB_PFCl_1 | -0.752 | 0.819 | 4.9 |
| lDefaultA_PFCm_1 | 1.04 | 0.975 | 4.7 |
| rDefaultB_PFCd_1 | 0.842 | 0.945 | 4.7 |
| rSomMotA_4 | -0.909 | 0.885 | 4.7 |
| lSomMotB_S2_1 | -4.13 | 3.23 | 4.6 |
| rContC_Cingp_1 | 0.136 | 3.73 | 4.6 |
| lDefaultA_pCunPCC_1 | 2.28 | 1.94 | 3.9 |
| rVisCent_ExStr_2 | -0.757 | 0.901 | 3.9 |
| lContC_Cingp_1 | -0.729 | 1.96 | 2.8 |
| lVisCent_ExStr_1 | -1.07 | 0.742 | 2.5 |
| lContA_PFCl_2 | -0.584 | 1.45 | 2.4 |
| lLimbicB_OFC_1 | 0.488 | 0.408 | 2.3 |
| rDefaultC_Rsp_1 | -1.39 | 1.28 | 2.3 |
| lDefaultB_PFCd_1 | -1.15 | 1.02 | 2.2 |
| rSomMotA_1 | 5.27 | 4.82 | 2.2 |
| rDefaultB_PFCv_2 | 1.16 | 1.02 | 2.1 |
| rSalVentAttnA_ParMed_1 | 0.689 | 0.669 | 2.1 |
| lContA_PFCl_1 | -1.12 | 0.98 | 1.9 |
| lDorsAttnB_PostC_1 | 0.79 | 0.697 | 1.9 |
| lVisPeri_StriCal_1 | 0.832 | 1.05 | 1.9 |
| lDorsAttnA_TempOcc_1 | -1.42 | 0.998 | 1.7 |
| rDorsAttnA_SPL_1 | 0.902 | 0.614 | 1.7 |
| lSalVentAttnA_ParMed_1 | -0.939 | 1.76 | 1.6 |
| lDefaultC_Rsp_1 | 0.59 | 3.04 | 1.5 |
| lSomMotA_2 | -0.0289 | 0.832 | 1.5 |
| rVisCent_ExStr_1 | 1.53 | 1.4 | 1.5 |
| rSalVentAttnA_ParOper_1 | 0.448 | 1.13 | 1.4 |
| rContA_PFCl_2 | 0.963 | 0.717 | 1.3 |
| rDorsAttnA_ParOcc_1 | 0.453 | 0.44 | 1.3 |
| rDorsAttnA_TempOcc_1 | -0.795 | 0.689 | 1.3 |
| rContB_PFCld_1 | -0.405 | 0.243 | 1 |

Note: clinical variables are highlighted in bold.

**Supplementary analyses – possible effects of MR sequence types**

Given that different MR sequence types were employed (i.e., T1-weighted and FLAIR), we replicated the analyses of Model 1 and Model 2 integrating MRI type (binary variable), to control for possible differences associated to the different kind of sequences.

The addition of the latter does not lead to increase in predictive ability of the models

- Model 1: R² = 0.581 ± 0.014 ; RMSE = 14.555 ± 0.245
- Model 1 with sequence type: R² = 0.575 ± 0.015; RMSE = 14.66 ± 0.255
- Model 2: R² = 0.57 ± 0.031; RMSE = 14.752 ± 0.521
- Model 2 with sequence type: R² = 0.567 ± 0.03; RMSE = 14.798 ± 0.516

In terms of selection of brain areas of Model 2, the addition of sequence type did not substantially affect the frequency of selection of the areas (see Tables S2 and S3, reporting brain the areas selected with a frequency > 50%):

**Table S2. Most frequently selected brain areas for Model 2**

| **Predictor** | **Mean Coeff** | **SD**  **Coeff** | **Frequency of selection** |
| --- | --- | --- | --- |
| lSalVentAttnA_ParOper_1 | 7.42 | 1.71 | 100 |
| lContA_IPS_1 | 6.89 | 1.96 | 99.8 |
| lVisPeri_ExStrInf_1 | -5.03 | 2.08 | 98.3 |
| rDorsAttnB_PostC_2 | 3.47 | 1.67 | 97.2 |
| lSomMotA_1 | 2.7 | 1.62 | 91.6 |
| rDorsAttnB_FEF_1 | -1.87 | 1 | 89.8 |
| lDefaultB_PFCv_1 | -2.38 | 1.42 | 89 |
| lDorsAttnA_ParOcc_1 | -2.31 | 1.52 | 83.4 |
| rDefaultA_PFCd_1 | -2.3 | 1.75 | 80.4 |
| lContC_pCun_1 | -3.72 | 3 | 65.2 |
| rContC_pCun_1 | -2.17 | 1.61 | 62.5 |
| lDefaultB_IPL_1 | 0.853 | 0.689 | 53.1 |
| rDefaultA_pCunPCC_1 | -1.77 | 1.69 | 52.2 |

**Table S3. Most frequently selected brain areas for Model 2 + MRI sequence type**

| **Predictor** | **Mean Coeff** | **SD**  **Coeff** | **Frequency of selection** |
| --- | --- | --- | --- |
| lSalVentAttnA_ParOper_1 | 7.39 | 1.71 | 100 |
| lContA_IPS_1 | 6.62 | 1.91 | 99.5 |
| lVisPeri_ExStrInf_1 | -5.12 | 2.11 | 98.3 |
| rDorsAttnB_PostC_2 | 3.34 | 1.64 | 96.3 |
| lSomMotA_1 | 2.7 | 1.6 | 90.9 |
| rDorsAttnB_FEF_1 | -1.9 | 1.01 | 89 |
| lDefaultB_PFCv_1 | -2.35 | 1.42 | 88.6 |
| lDorsAttnA_ParOcc_1 | -2.25 | 1.54 | 81.8 |
| rDefaultA_PFCd_1 | -2.23 | 1.74 | 77.8 |
| lContC_pCun_1 | -3.65 | 3.01 | 65.8 |
| rContC_pCun_1 | -2.07 | 1.56 | 60.9 |
| lDefaultB_IPL_1 | 0.868 | 0.687 | 50.8 |

**References**

1. Kirshblum SC, Burns SP, Biering-Sorensen F, et al. International standards for neurological classification of spinal cord injury (revised 2011). *J Spinal Cord Med*. Nov 2011;34(6):535-46. doi:10.1179/204577211X13207446293695

2. Iglesias JE, Billot B, Balbastre Y, et al. SynthSR: A public AI tool to turn heterogeneous clinical brain scans into high-resolution T1-weighted images for 3D morphometry. *Sci Adv*. Feb 3 2023;9(5):eadd3607. doi:10.1126/sciadv.add3607

3. Iglesias JE, Billot B, Balbastre Y, et al. Joint super-resolution and synthesis of 1 mm isotropic MP-RAGE volumes from clinical MRI exams with scans of different orientation, resolution and contrast. *Neuroimage*. Aug 15 2021;237:118206. doi:10.1016/j.neuroimage.2021.118206

4. Baldi S, Chiulli N, Palm S, et al. Targeting depression circuitry with H1 coil Transcranial Magnetic Stimulation: a retrospective circuit mapping study. *Neuropsychopharmacology*. Oct 2025;50(11):1674-1682. doi:10.1038/s41386-025-02157-5

5. Achiron A, Warszawer Y, Nissan Y, et al. Positive impact of cladribine tablets on reducing brain atrophy in patients with relapsing-remitting multiple sclerosis: A longitudinal study. *Mult Scler*. May 2025;31(6):689-695. doi:10.1177/13524585251313749

6. Gaser C, Dahnke R, Thompson PM, Kurth F, Luders E, The Alzheimer's Disease Neuroimaging I. CAT: a computational anatomy toolbox for the analysis of structural MRI data. *Gigascience*. Jan 2 2024;13doi:10.1093/gigascience/giae049

7. Ashburner J, Friston KJ. Diffeomorphic registration using geodesic shooting and Gauss-Newton optimisation. *Neuroimage*. Apr 1 2011;55(3):954-67. doi:10.1016/j.neuroimage.2010.12.049

8. Diana L, Sritharan J, Vallesi V, Brunello N, Verma R, Zito G. Chronic spinal cord injury is associated with morphometric brain changes in functional networks beyond the sensorimotor system. *Frontiers in Neurology*. 2025;

9. Yeo BT, Krienen FM, Sepulcre J, et al. The organization of the human cerebral cortex estimated by intrinsic functional connectivity. *J Neurophysiol*. Sep 2011;106(3):1125-65. doi:10.1152/jn.00338.2011
